# Valorizing Southeast Asian agricultural residues into high-performance mycelium-based biocomposites

**DOI:** 10.1038/s41598-026-66400-9

**Published:** 2026-08-13

**Authors:** Alireza Javadian, Xin Ying Chan, Nazanin Saeidi, Dirk E. Hebel, Frank Schultmann

**Affiliations:** 1 Faculty of Architecture, Karlsruhe Institute of Technology (KIT), Englerstrasse 11, 76131 Karlsruhe, Germany; 2 Institute for Industrial Production (IIP), Karlsruhe Institute of Technology (KIT), Hertzstraße 16, 76187 Karlsruhe, Germany

**Keywords:** Mycelium composites, Bio-based materials, Agricultural residues, Waste valorization, Biotechnology, Environmental sciences, Plant sciences

## Abstract

The increasing generation of waste is a global challenge with particularly severe impacts in Southeast Asia, where agricultural and industrial lignocellulosic residues are often underutilized or inadequately managed. Major waste streams include sugarcane bagasse, *Albizia chinensis* woodchips, and cassava pulp. This study demonstrates that these abundant residues can be effectively valorized as feedstocks for the production of high-performance mycelium-based composite materials with an average density of approximately 365 kg/m^3^. Sugarcane bagasse and *Albizia chinensis* woodchips serve as effective base substrates supporting stable mycelial growth of *Ganoderma lucidum*, while cassava pulp functions as a nutrient-rich carbohydrate supplement that influences composite microstructure and mechanical performance. Albizia woodchip substrates supplemented with cassava pulp exhibited the highest compressive strength, reaching 0.63 MPa at 5% strain. These findings demonstrate that substrate composition plays a key role in determining the microstructure and mechanical performance of mycelium composites, providing a viable pathway for transforming underutilized lignocellulosic waste into functional bio-based materials.

## Introduction

The extraction, use, and disposal of materials have become significant global challenges for both resource security and environmental sustainability. Municipal solid waste generation is projected to increase from approximately 2.1 billion tonnes in 2023 to nearly 3.8 billion tonnes by 2050, with more than one-third of this waste expected to be openly dumped or burned^1,2^. Such disposal practices release greenhouse gases and fine particulate matter (PM_2.5_), degrade soil and water quality, and contribute to climate change and adverse public-health impacts worldwide^3,4^.

Southeast Asia (SEA) is among the regions most severely affected by these waste and pollution burdens. Agricultural and industrial residues are frequently disposed of through open burning, contributing substantially to poor regional air quality^5,6^. In Vietnam, approximately 50 million tonnes of crop residues are generated annually, and poorly managed burning of these crop residues has been linked to economic losses of up to €14.3 billion annually, driven by raw material losses and adverse health impacts^7^. Indonesia, Thailand, and Cambodia report similar burning patterns, where open combustion remains the lowest-cost disposal method^8^. Biomass burning in the region can increase PM_2.5_ concentrations by tens of µgm^-3^ during the dry season and has been linked to hundreds to thousands of premature deaths annually^9^.

Valorizing agricultural residues by converting them into value-added materials offers a promising strategy for reducing open burning while strengthening regional circular economies. Such approaches can reduce the environmental burdens associated with current disposal practices while creating new opportunities for sustainable construction and furniture materials.

Fungal mycelium provides a natural and biological route to transform regionally abundant agricultural residues into engineered, fully bio-based composites. Mycelium, the filamentous vegetative network of fungi, forms a natural binding matrix that colonizes and consolidates plant-based substrates. Through extracellular enzyme secretion, including oxidoreductases such as laccase, lignin peroxidase, and manganese peroxidase, the mycelium primarily modifies lignin, while hemicellulose is degraded mainly by hydrolases such as glycoside hydrolases and esterases. Together, these enzymatic processes enable progressive colonization of the lignocellulosic substrate and the formation of a cohesive mycelium-bound composite without the use of synthetic binders^10,11^. This biologically driven process produces lightweight and biodegradable composites with tunable density and mechanical properties. The tropical climate in SEA matches the temperature and humidity conditions required for fungi growth, requiring only limited external energy input for cultivation. As a result, mycelium-based materials have been explored for applications such as packaging, insulation, acoustic panels, and lightweight structural components, demonstrating their potential as sustainable alternatives that contribute to a circular bioeconomy and a low-carbon construction sector^12–14^.

Despite significant progress in laboratory-scale demonstrations, industrial adoption of mycelium-based composites remains limited, primarily due to challenges in process control and material consistency. The growth rate and final mechanical properties of fungal composites are influenced by environmental and processing parameters such as temperature, humidity, aeration, particle size, nutrient composition, and compaction pressure^11,15–18^. The substrate’s physical and chemical characteristics govern both colonization kinetics and the resulting density of the mycelial network, which in turn affects the composite’s stiffness and strength^18,19^. Substrate selection is therefore a critical design parameter for achieving reliable and scalable material properties tailored for specific applications.

Traditionally, substrates such as hemp hurds, flax shives, and cereal straw have been widely used for mycelium composites because of their porous structure and balanced nutrient content^20–22^. However, these substrates are not readily available in regions around the world, and reliance on non-local feedstocks increases both material costs and the associated environmental footprint. The utilization of locally available agricultural and industrial by-products offers a more sustainable approach by minimizing transportation-related impacts, valorizing regional waste streams^23^, and creating opportunities for substantial economic benefits through value-added product development^24^.

Three lignocellulosic residues were selected as substrates for fungal growth: *Albizia chinensis* woodchips (AWC), sugarcane bagasse (SC), and cassava pulp. These materials were chosen because they represent abundant agricultural and industrial by-products in Southeast Asia that remain underutilized or disposed of through open burning, contributing to regional air pollution and greenhouse gas emissions^25–28^.

The first residue stream, SC, is a by-product of the sugar production process. It accounts for approximately 30% of the mass of harvested raw sugarcane processed. Indonesia’s annual sugarcane production of approximately 33 million tonnes, therefore, generates approximately 10 million tonnes of bagasse. Comparable volumes are produced in Thailand and the Philippines, where large, centralized sugar mills operate year-round^29,30^. SC contains approximately 40–45% cellulose, 25–30% hemicellulose, and 20–25% lignin on a structural dry-weight basis^31^, which white rot fungi can enzymatically degrade to sustain growth. In addition, studies have reported the release of up to 0.4 g/g of soluble sugars (including sucrose, glucose and fructose), which may also promote initial mycelial colonization.

The second residue stream, cassava pulp, is generated as a by-product of starch extraction from fresh cassava roots. Thailand processes approximately 33 million tonnes of cassava annually, while Lampung province in Indonesia hosts a concentrated cluster of cassava processing factories and is among the country’s leading cassava-producing regions^32,33^. Together, these regions generate substantial quantities of cassava pulp, providing an abundant feedstock for mycelium-based composite production. Despite being considered a low-value by-product, cassava pulp contains 30–80% residual starch, which serves as a readily available nutrient source for fungal metabolism, and promotes rapid mycelial colonization^34,35^.

Wood processing contributes a third major residue stream, with sawmills and panel mills typically generating around 40–50% residues relative to input wood volume^28,36^. Fast-growing plantation species commonly cultivated for plywood and other wood-based panel production include *Albizia chinensis* and *Paraserianthes falcataria*. Processing these species for furniture, plywood, and particleboard applications generates residual chips and fines^37,38^. *Albizia chinensis* woodchips from sawmills and plywood industries contain 40–50% cellulose, 15–25% hemicellulose and 20–30% lignin, providing structurally rigid fibers that support fungal adhesion and the formation of an interconnected mycelial network^39–41^.

Together, these three industries generate a large, regionally concentrated reservoir of lignocellulosic biomass across Indonesia, Thailand, and the Philippines. Repurposing even a small portion of this underutilized feedstock into engineered bio-based composites offers environmental and economic benefits. The benefits of valorizing agricultural residues include reducing open-burning emissions and waste accumulation, lowering dependence on imported wood and fossil-derived materials, and establishing region-specific circular value chains. These value chains support the development of sustainable materials for construction, furniture, and interior applications within the SEA region.

Building on these material characteristics, this study investigates the feasibility of utilizing these locally abundant by-products, either individually or in combination, as substrates for the white-rot fungus *Ganoderma lucidum*. By systematically linking substrate composition, fungal growth behaviour, and resulting mechanical properties, this work advances our understanding of their relationship. This study demonstrates a pathway for the conversion of regional waste streams into functional bio-based materials suited for construction and furniture applications in tropical contexts.

Many fungal species have been studied for mycelium-based composites, each exhibiting different metabolic pathways and hyphal architectures. White-rot fungi, such as *Pleurotus*, *Trametes*, and *Ganoderma* are particularly suitable, as they degrade lignin while modifying cellulose and hemicellulose with their diverse set of lignin-modifying enzymes, including laccases, lignin peroxidases, and manganese peroxidases, enabling efficient colonization of lignocellulosic materials^10,11,42^. *G. lucidum*, particularly, produces dense and well-bonded mycelial networks, and is widely employed for composite fabrication due to its enzymatic versatility and strong fiber-binding capability^7,8^. The broad enzymatic capacity to modify the major cell wall polymers is associated with more uniform bonding between substrate particles, which may contribute to improved structural integrity in the resulting composites.

*G. lucidum* is a trimitic fungus, meaning its hyphal system consists of three types of hyphae: generative, skeletal, and binding hyphae. Skeletal and binding hyphae are typically thick-walled and contribute to the rigidity and mechanical strength of the mycelial network^43^. Compared with commonly used monomitic fungi such as *Pleurotus*, which possess only generative hyphae, the trimitic hyphal system of *G. lucidum* is therefore expected to provide a mechanically robust mycelial network. In addition, *G. lucidum* tolerates a wide range of humidity and temperature conditions, and has been successfully used in prior studies on mycelium-based composites for its consistent morphology, predictable growth kinetics, and reliable mechanical performance^19,44^. Together, these characteristics make *G. lucidum* a suitable fungal species for developing bio-based composites from agricultural and industrial residues as sustainable raw materials available in tropical regions.

While mycelium composites are typically cultivated as a lightweight, foam-like material, they can exhibit sufficient rigidity for self-supporting and non-load-bearing applications ^45^. Camilleri et al. highlighted that improving the mechanical performance of mycelium-based composites is a key step toward expanding their applications across a broad range of industries. The processing strategy investigated in this study contributes to this objective by examining how substrate selection and nutrient supplementation influence the composite microstructure and mechanical performance^46^. Previous studies have explored post-growth densification techniques, such as hot and cold pressing after mycelium deactivation to improve the mechanical properties, but these approaches present several limitations^19,47^. While hot pressing can enhance strength through thermally induced lignin bonding, it is energy-intensive^23^. Cold pressing, in contrast, generally leads to limited improvement in material strength due to disruption of the existing mycelial network without the presence of heat-induced re-bonding. Here, we therefore propose an alternative approach in which the substrate is compressed during active mycelial growth. This mid-growth compression approach allows the compressed material to undergo further fungal growth and consolidation while avoiding the high energy requirements of hot pressing and using regionally available agricultural by-products.

In this study, three locally available residues were selected to evaluate their suitability as substrates for *G. lucidum.* Both AWC and SC successfully served as base substrates for growing mycelium-based composites. The addition of cassava pulp to the base substrates produced contrasting effects depending on the base substrate, substantially increasing the compressive strength and modulus of AWC-based composites while reducing both properties in SC-based composites. The results indicate that substrate composition is a key factor influencing the development of mycelium-based composites, as it influences substrate-hypha interactions and, consequently, the mechanical performance of the resulting material.

## Results

### Suitability of substrates for mycelium growth

Mycelium growth was achieved on both AWC and SC-based substrates, both with and without cassava pulp. Using the three-stage growth and mid-growth compression approach, all formulations produced dense mycelium composites. Across all formulations, the dried composites had an average density of 365 ± 84 kg/m^3^, approximately twice that of naturally packed mycelium foams prepared with similar substrates^48,49^.

Mycelial growth initiated uniformly from the inoculation points and spread through the substrate matrix during the first incubation phase. Both AWC and SC substrates supported continuous mycelial colonization, with the three-stage growth process completed within 10–20 days, depending on the substrate formulation. Visual observations showed that hyphal strands initially filled the pore spaces between substrate particles and subsequently formed a cohesive, continuous network, consistent with the established colonization pattern of *G. lucidum*.

**Fig. 1:**
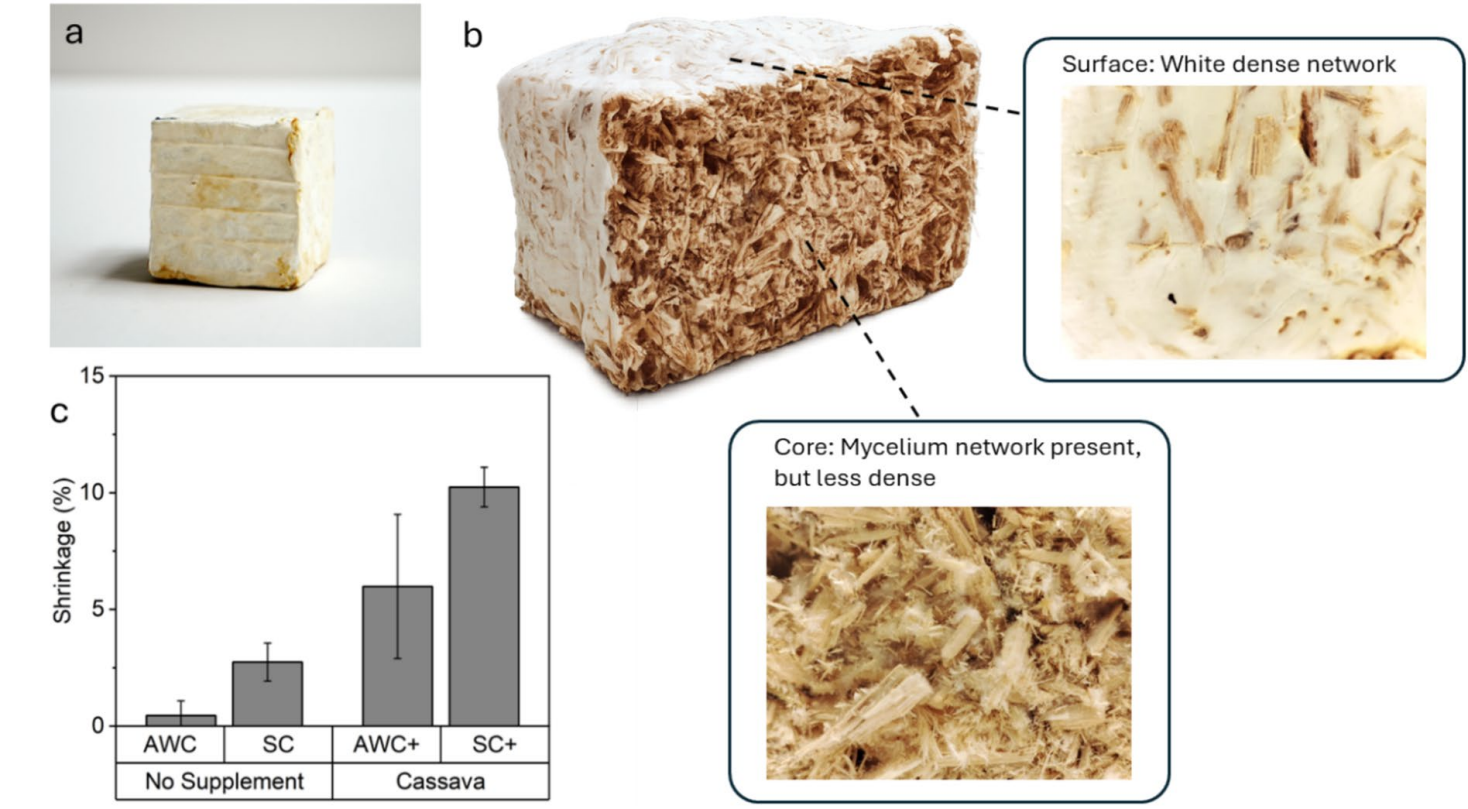
Macroscopic appearance and dimensional stability of mycelium composites. The mycelium composites are covered by a uniform, continuous white mycelial skin that remains intact following shrinkage induced by drying. (**a**) Fully grown and dried sample, with an even, white continuous layer of mycelium skin, (**b**) Observable differences between mycelium growth on sample surfaces, where the mycelium forms a dense white network, and in the core of the sample, where mycelium network is less dense. (**c**) Drying-induced shrinkage of mycelium composites produced from AWC and SC, comparing samples without supplementation and those supplemented with cassava pulp.

At the end of the growth process, all samples developed a white, continuous mycelial skin approximately 1–2 mm thick, indicating complete surface colonization (Fig. 1a).

This outer surface consisted of a dense, highly interconnected network of aerial mycelial hyphae, giving the composite its characteristic white appearance (Fig. 1b). In contrast, the core remained dominated by the lignocellulosic substrate and exhibited a less densely interconnected mycelial network. Although mycelium was present throughout the core and contributed to binding the substrate particles, its lower density allowed the underlying brown lignocellulosic substrate to remain visible.

The consistent formation of a uniform outer skin and successful internal colonization across all samples demonstrates the suitability of these tropical residues for producing mycelium-based composites.

### Effects of substrate composition on drying-induced shrinkage

Oven drying induced measurable dimensional changes in all composites, with the extent of shrinkage depending on both the base substrate type and the addition of cassava. Composites produced from AWC without cassava supplementation exhibited the lowest shrinkage (0.4%), whereas SC-based composites without cassava showed a shrinkage of 2.7% following oven drying (Fig. 1c). Cassava supplementation substantially increased shrinkage in both substrate systems, reaching 6.0% in AWC-based composites and 10.2% in SC-based composites (Fig. 1c). A two-way ANOVA, followed by Tukey’s post hoc multiple comparison test, showed that both substrate type and cassava supplementation had statistically significant effects on drying-induced shrinkage (*p* < 0.05), whereas no significant interaction between the two factors was observed (*p* > 0.05). These findings indicate that substrate type and cassava supplementation independently influenced drying-induced shrinkage, with SC-based composites exhibiting greater shrinkage than AWC-based composites and cassava supplementation consistently increasing shrinkage in both substrate types.

### Microstructural characteristics of composites

**Fig. 2:**
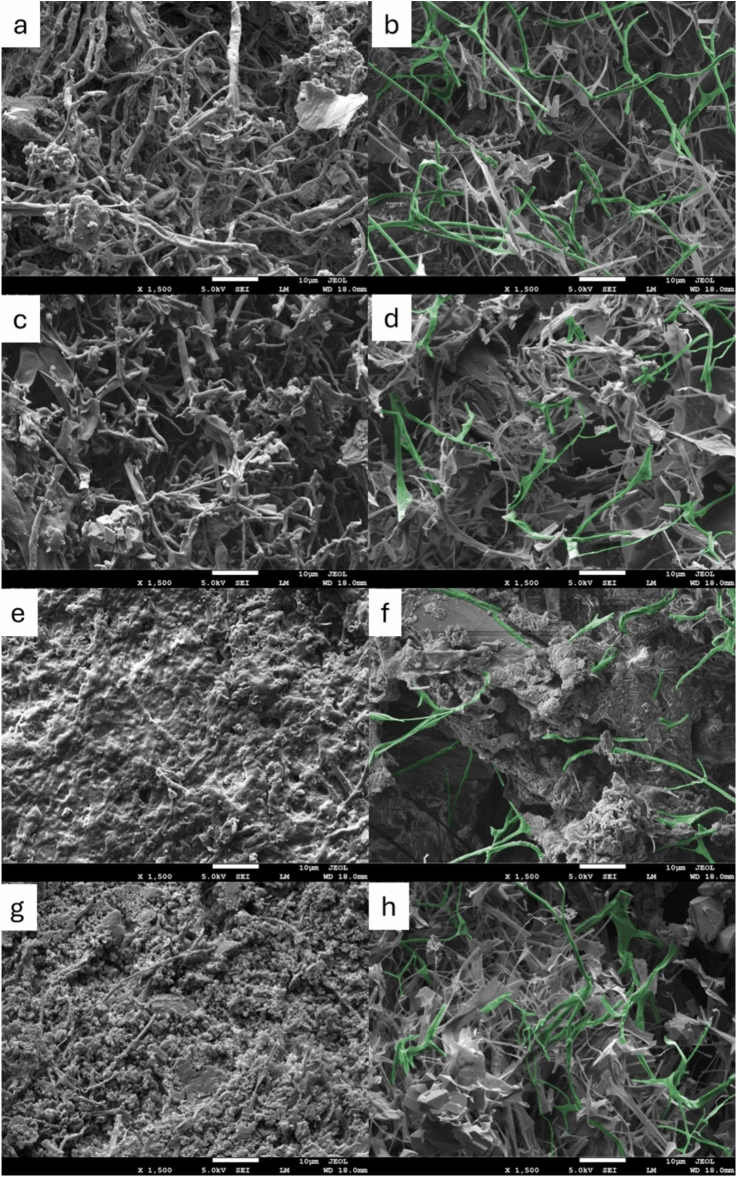
SEM images showing the microstructural morphology of mycelium composites produced from different substrate formulations. (**a**) The surface (skin) of an AWC-based composite, (**b**) core of an AWC-based composite, (**c**) surface (skin) of an SC-based composite, d) core of an SC-based composite. Both AWC- and SC-based composites without cassava exhibited similar hyphal network morphologies in the surface and core regions. (**e**) Surface (skin) of an AWC-based composite supplemented with cassava pulp, (**f**) core of an AWC-based composite supplemented with cassava pulp, (**g**) surface (skin) of a SC-based composite supplemented with cassava pulp, and (**h**) core of a SC-based composite supplemented with cassava pulp. Cassava-supplemented composites exhibited a distinct granular surface morphology while retaining an underlying interconnected network containing representative filamentous structures consistent with fungal hyphae, highlighted in green in the core regions (**b**, **d**, **f**, **h**).

Scanning Electron Microscopy (SEM) was used to examine the influence of substrate composition and cassava supplementation on the hyphal network and pore architecture of the composites. Figure 2a, b show the morphology of AWC-based samples without cassava, while Fig. 2c, present SC-based samples without cassava. Both substrates exhibited dense and continuous hyphal growth on the external surface (skin) and internal regions (core). The interconnected hyphal network formed a coherent surface layer across the AWC and SC fiber surfaces. Hyphae closely enveloped particle edges and bridged adjacent particles, filling local pore spaces and reinforcing particle contacts. The core region of AWC-based composites (Fig. 2b) exhibited similarly uniform colonization, indicating comparable hyphal growth in both surface (skin) and core regions.

SC-based composites exhibited a similar fibrous microstructure to AWC- based composites. The surface (skin) region (Fig. 2c) showed elongated hyphae bridging the slender SC fibers, forming a continuous network similar to that observed in the AWC-based composites, although with a lower fiber density. The core region (Fig. 2d) exhibited a comparable hyphal network morphology to the surface region, with clearly identifiable hyphae distributed throughout the substrate, indicating homogeneous colonization across the composite thickness. The similar hyphae network structures observed in both the surface and core regions of the AWC- and SC-based composites suggest that the mid-growth compression method did not prevent mycelial colonization throughout the composite thickness.

The incorporation of cassava produced distinct microstructural features in all supplemented samples (Fig. 2e–h). In AWC samples supplemented with cassava (Fig. 2e), the surface morphology was characterized by a dense granular texture superimposed on the fibrous hyphal network. A similar granular texture was observed on the surface of SC samples supplemented with cassava (Fig. 2g). These granules were concentrated mainly on the composite surface but were also present within the core regions (Fig. 2f, h), where the underlying hyphal network remained clearly visible. As these granular features were observed only in the cassava-supplemented formulations, they are likely associated with the presence of residual cassava-derived fines.

Overall, substrate composition had little influence on the morphology of the hyphal network, and all samples without cassava exhibited similar structures in both the surface and core regions. In contrast, cassava supplementation introduced distinct granular features, and the core regions of cassava-supplemented composites also exhibited noticeable morphological differences compared with the non-supplemented composites, suggesting changes in theinternal organization of the substrate-hypha network which are further discussed in the following section.

### Mycelial fiber size distribution

To assess whether substrate type or cassava supplementation affected the hyphal morphology of *G. lucidum*, hyphal diameters were measured from SEM images of the core regions of the composites (Fig. 2b, d, f, h). Only the core morphology was analysed in this study, as the surface layers formed dense mycelial mats in which individual hyphae could not be consistently distinguished. In contrast, the core regions displayed clearly distinguishable hyphal strands with well-defined boundaries, enabling consistent and reproducible diameter measurements.

**Fig. 3:**
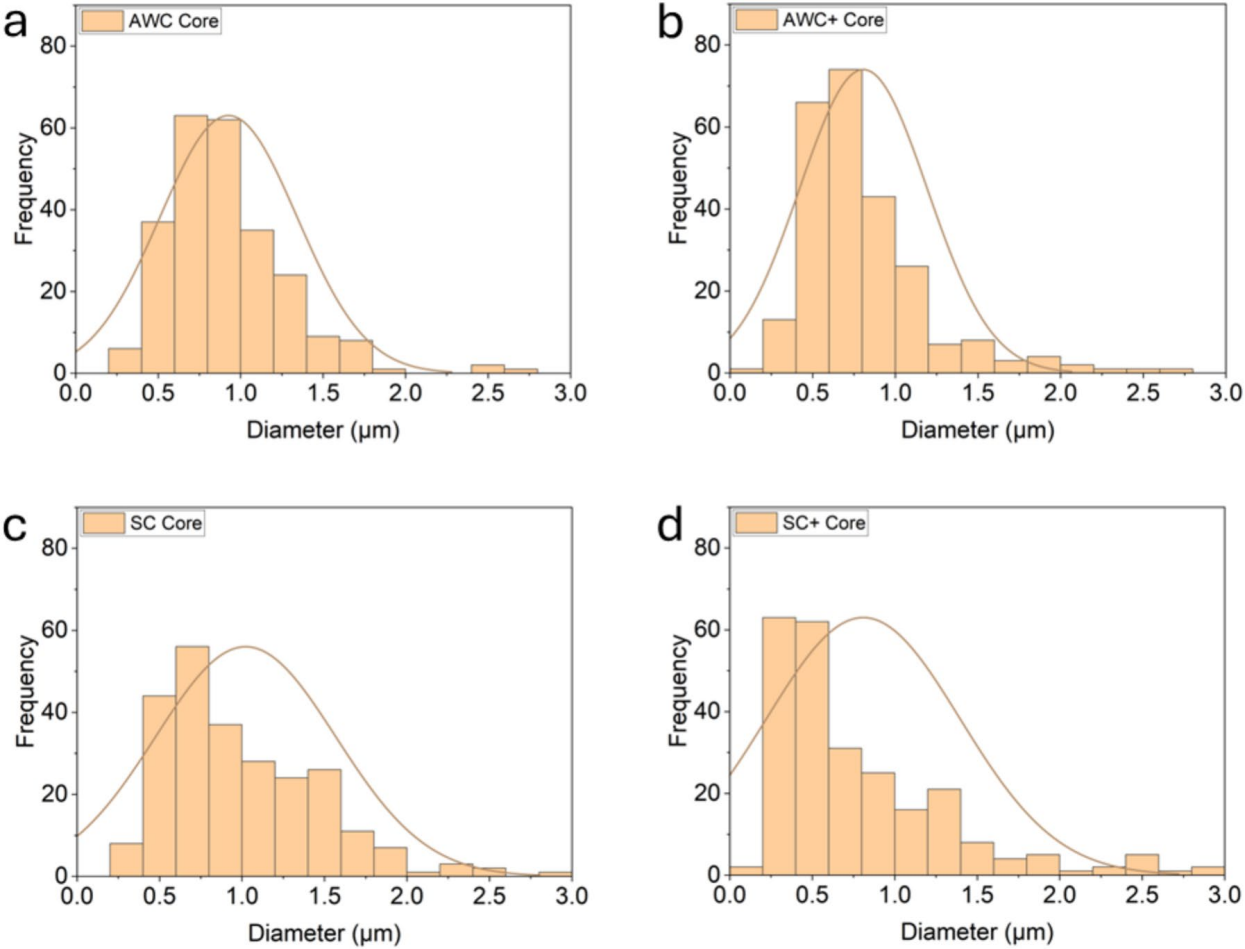
Mycelial diameter distribution in mycelium composites produced from different substrates. Mycelial hyphal diameter distributions for the core of (**a**) AWC-based composites, (**b**) AWC-based composites supplemented with cassava, (**c**) SC-based composites, and d) SC-based composites supplemented with cassava. Smooth curves represent the fitted normal distribution curves for each dataset.

For composites produced without cassava supplementation, the mean hyphal diameters were 0.93 ± 0.42 µm for AWC-based composites and 1.02 ± 0.55 µm for SC-based composites (Fig. 3a, b). The hyphal diameter distributions were similar and centred around 1 µm, indicating that the *G. lucidum* maintained a similar hyphal morphology across both substrates. This observation is consistent with the SEM image analysis, which showed uniform colonization and comparable hyphal network structures throughout the core regions of the AWC- and SC-based composites in the absence of cassava supplementation.

Cassava supplementation led to a clear shift towards finer hyphae in both substrate types. AWC-based composites supplemented with cassava exhibited a mean hyphal diameter of 0.81 ± 0.39 µm, while SC-based composites supplemented with cassava showed a similar mean diameter of0.81 ± 0.59 µm (Fig. 3c, d). A two-way ANOVA followed by Tukey’s post-hoc multiple comparison test showed that cassava supplementation had a statistically significant effect on hyphal diameters (*p* < 0.05), whereas the substrate type (AWC versus SC) did not have a statistically significant effect (*p* > 0.05).

### Mechanical performance of the composites

**Fig. 4:**
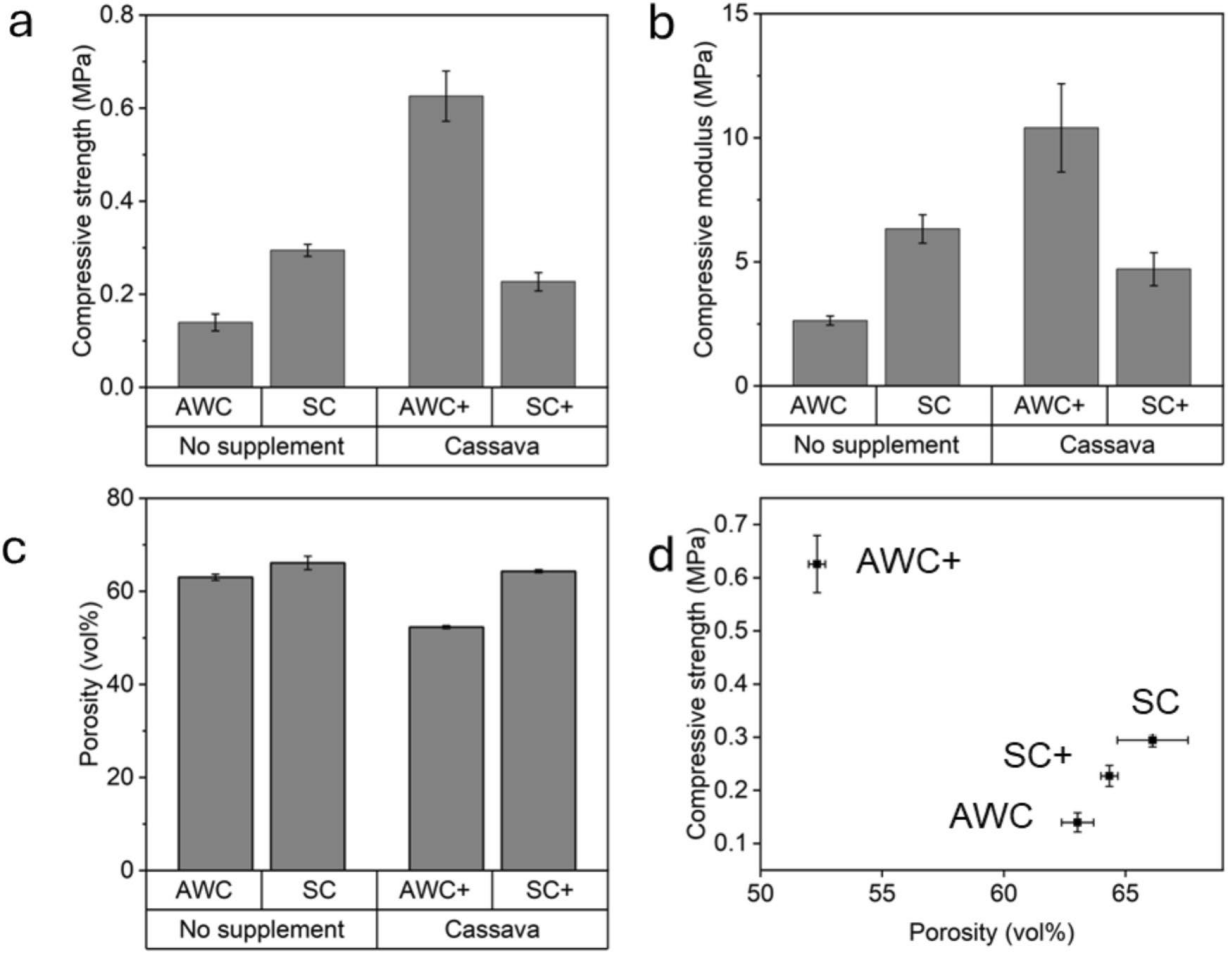
Mechanical performance and porosity of mycelium composites produced from different substrate formulations. (**a**) Compressive strength measured at 5% strain, (**b**) compressive modulus, (**c**) porosity, (**d**) compressive strength plotted as a function of porosity for all formulations. Error bars represent the standard deviation of the measurements.

The choice of substrate and cassava supplementation strongly influenced the mechanical properties of the mycelium composites. Figure 4. summarizes the compressive strength (measured at 5% strain), compressive modulus, porosity, and correlation between porosity and compressive strength for all formulations. These properties reflect how variations in hyphal network morphology, pore architecture, and substrate-hypha interactions translate into differences in mechanical performance as discussed in earlier sections. Among the unsupplemented composites, SC-based composites exhibited higher compressive strength and higher compressive modulus than AWC-based composites. AWC-based composites had a compressive strength of 0.14 ± 0.02 MPa and a compressive modulus of 2.6 ± 0.2 MPa (Fig. 4a, b). SC-based composites were approximately twice as strong and stiff, with a compressive strength of 0.29 ± 0.01 MPa and a compressive modulus of 6.3 ± 0.6 MPa.

Cassava supplementation produced contrasting effects on the two substrates. When cassava was added to AWC-based composites, the compressive strength increased nearly fourfold to 0.63 ± 0.05 MPa, while the compressive modulus increased to 10.4 ± 1.8 MPa, representing the highest mechanical performance among all formulations in this study.

In contrast, cassava supplementation led to a reduction in the mechanical properties of SC-based composites. Compressive strength decreased from 0.29 ± 0.03 MPa to 0.23 ± 0.02 MPa, while the compressive modulus decreased from 6.3 ± 0.6 MPa to 4.7 ± 0.7 MPa.

Two-way ANOVA followed by Tukey’s multiple comparison test showed that both compressive strength and compressive modulus were significantly affected by substrate type, cassava supplementation, and their interaction (p < 0.05 for all effects). These results indicate that the mechanical properties depended on both factors and that the effect of cassava supplementation varies between substrate types.

Overall, these findings demonstrate that the mechanical response to cassava supplementation was strongly substrate-dependent, substantially improving the mechanical performance of AWC-based composites, whereas SC-based composites exhibited a moderate reduction in compressive strength and modulus.

### Porosity

Figure 4c shows that the porosity of AWC and SC-based composites without cassava supplementation was similar, although AWC-based composites exhibited a slightly lower porosity (63.0 ± 0.7 vol%) than SC-based composites (66.1 ± 1.5 vol%). With cassava supplementation, the porosity of AWC-based composites decreased substantially to 52.3 ± 0.3 vol%, whereas the porosity of SC-based composites decreased only slightly to 64.3 ± 0.3 vol%. These results indicate that cassava supplementation affected the pore structure differently depending on the base substrate. As shown in Fig. 4d, the relationship between porosity and compressive strength differed among the formulations. In particular, the AWC-based composite supplemented with cassava exhibited the highest compressive strength despite having the lowest porosity, indicating that porosity alone does not account for the observed differences in mechanical performance.

## Discussion

Overall, these results show that AWC, SC, and cassava-supplemented mixtures all served as effective fungal substrates, but with distinct growth behaviours that contributed to differences in shrinkage and mechanical performance.

AWC-based composites exhibited lower shrinkage than SC-based composites, and the addition of cassava increased shrinkage in both types of composites. The observed differences in shrinkage are likely related to variations in the physicochemical properties of the substrate fibers. Lignocellulosic fibers, including AWC and SC, are inherently hydrophilic and undergo swelling when exposed to moisture^50^. However, AWC fibers generally exhibit lower dimensional changes than SC fibers due to differences in their cellular structure^51^. As these fibers constitute the majority of the composite matrix, their swelling and subsequent drying behaviour strongly influence the dimensional stability of the final material. Consequently, AWC-based composites exhibited lower shrinkage than SC-based composites after drying. Cassava pulp is highly porous and hydrophilic and can contain up to 80% moisture, which is considerably higher than that of typical lignocellulosic fibers^52^. The incorporation of cassava, therefore, increases the overall water-holding capacity of the substrate mixture, resulting in greater shrinkage during drying for both AWC and SC-based composites. While the precise interactions among substrate composition, moisture retention, mycelial growth, and drying behaviour are beyond the scope of the present study, further investigation of these mechanisms would support future scale-up and product development. Such knowledge could enable the optimization of mold geometries, drying schedules, and dimensional tolerances according to substrate composition and nutrient supplementation, particularly for applications where dimensional accuracy is critical.

SEM observations combined with hyphal diameter analysis provide insight into how substrate composition influences mycelial growth. In this study, differences between the AWC and SC substrates did not significantly affect the morphology of the hyphal network. Only the addition of cassava resulted in a distinct granular surface morphology and was associated with significantly smaller hyphal diameters. One possible explanation is that the high starch content in cassava pulp provided readily metabolizable sugars that altered fungal growth behaviour. Under nutrient-rich conditions, many white-rot fungi have been reported to produce faster-growing but thinner hyphae. Although fungal growth rate was not measured directly in the present study, the observed reduction in hyphal diameter following cassava supplementation is consistent with these previous observations^53,54^.

Interestingly, although cassava supplementation was associated with finer hyphae and distinct changes in microstructure, it did not necessarily result in higher composite strength. This suggests that the mechanical performance of mycelium composites depends on factors beyond hyphal morphology alone.

Substrate composition appears to play a critical role in determining mechanical behaviour. AWC-based composites exhibited lower strength than SC-based composites, which may be related to differences in substrate composition and nutrient availability. SC contains a greater proportion of readily metabolizable carbohydrates than AWC^55,56^, which may support fungal growth before the gradual degradation of lignocellulosic components.

Although these differences were not clearly evident in the SEM images, they were reflected in the compressive strength of the composites. The degradation of lignocellulosic materials by fungi requires a complex enzymatic process and therefore occurs more gradually than the utilization of simple sugars. As SC contains both lignocellulosic components and readily accessible sugars, it may provide a more favourable nutritional environment during the early stages of fungal colonization than AWC alone.

However, the effect of cassava supplementation differed between the two substrates. While the addition of cassava reduced the strength of SC-based composites, AWC composites supplemented with cassava exhibited the highest compressive strength among all formulations. This may be attributed to the high starch content of cassava pulp, which provides an accessible nutrient source that complements the structural lignocellulosic components of AWC, thereby supporting improved bonding between the fungal network and substrate particles. In contrast, SC already contains a substantial fraction of accessible sugars; therefore, additional cassava may have reduced the proportion of structural fibers within the substrate mixture without providing a corresponding benefit to network formation, resulting in lower composite strength. These findings suggest that achieving an appropriate balance between nutritional content and structural components is critical for optimizing the mechanical performance of mycelium composites.

Porosity generally decreased with cassava supplementation, particularly in the AWC-based composite. SEM observations suggest that this reduction is associated with the presence of granular cassava particles occupying part of the void space within the composite structure. However, the relationship between porosity and compressive strength was not straightforward. Among the samples from AWC, SC, and SC supplemented with cassava formulation, higher porosity was associated with higher compressive strength. In contrast, the AWC-based composite supplemented with cassava exhibited the highest compressive strength despite having the lowest porosity. These results indicate that porosity alone does not govern the mechanical performance of the composites, and that additional factors, including substrate composition and substrate-hypha interactions, also contribute to the observed behaviour.

However, the AWC composite supplemented with cassava exhibited the highest compressive strength despite having the lowest porosity among all formulations. These results indicate that porosity alone does not govern composite strength. Additional factors, including substrate-hypha interactions and the extent of mycelial bonding observed in the SEM images, are likely to contribute to the mechanical performance of the composites.

To evaluate the performance of the developed biocomposites, their mechanical properties were compared with those reported in recent studies on mycelium-based materials tested under similar strain ranges (≤ 20%). As shown in Table 1, the material developed in this study achieved a compressive strength of 0.63 MPa at 5% strain with a density of approximately 365 kg/m^3^. When compared with previously reported mycelium-based composites, which typically exhibit compressive strengths ranging from 0.0031 to 0.155 MPa at comparable strain levels, the present material exhibits a marked improvement in strength and a favourable combination of high compressive strength and relatively low density. In the context of this study, the term “high-performance” refers to this combination of relatively low density and high compressive strength compared with comparable mycelium-based composites reported in the literature.

**Table 1:** Comparison of the density and compressive strength of representative mycelium-based materials reported in recent studies using comparable compression strain ranges (5–20%).

Density (kg/m^3^)	Strength (MPa)	Corresponding strain (%)	References
365	0.63	5	This study
31	0.0031	10	^57^
552	0.1459	20	^58^
114	0.155	10	^59^
122	0.128	15	^60^
170	0.1	10	^61^

Taken together, these results demonstrate that agricultural residues from Southeast Asia can be effectively valorized for the production of mycelium composites with enhanced mechanical performance. As discussed above and summarized in Table 1, the developed composites exhibited a favourable combination of relatively low density and high compressive strength compared with comparable mycelium-based composites reported in the literature. Studies by De et al. and Jones et al. have reported promising thermal insulation and fire-resistance for mycelium-based materials^62,63^, which are important properties for applications in the building and construction industry. Although challenges remain regarding water absorption and long-term durability, Elsacker et al. showed that densification through compression can reduce water uptake by limiting water diffusion pathways within the material^21^, while protective coatings have also been reported to significantly improve the durability of mycelium composites under moist and warm conditions^48^. These findings, combined with the mechanical performance demonstrated in the present study, indicate that mycelium composites derived from regional agricultural residues have strong potential as sustainable materials for the construction and furniture industries.

## Conclusion

This study demonstrates that abundant agricultural and industrial residues from Southeast Asia, including sugarcane bagasse, Albizia chinensis woodchips, and cassava pulp, can be effectively valorized into mycelium-based composites with promising mechanical performance. Both substrate composition and cassava supplementation significantly influence the microstructure, porosity, and mechanical properties of the composites. In particular, cassava supplementation substantially improved the compressive strength and modulus of AWC-based composites, while producing a different response in SC-based composites, highlighting the substrate-dependent nature of mycelium composite development.

The results further demonstrate that porosity alone does not determine the mechanical performance of the composites. Although cassava supplementation generally reduced porosity, the relationship between porosity and compressive strength was not straightforward, indicating that additional factors, including substrate-

hypha interactions and the extent of mycelial bonding between substrate particles, also contribute to the final material properties. Further chemical and microstructural characterization will provide deeper insight into the mechanisms governing these interactions.

Overall, the mid-growth compression method proved effective for producing rigid mycelium composites from agricultural residues. Beyond the material development presented here, this work highlights the potential of converting regionally abundant waste streams into functional biobased materials through low-energy manufacturing approaches. Such an approach can reduce the environmental burdens associated with current disposal practices while enabling decentralized, low-energy manufacturing rooted in local resource cycles. Collectively, these findings provide a practical framework for valorizing tropical biomass and advancing sustainable, region-specific alternatives for future construction and furniture applications.

## Methods

### Substrate materials

Albizia chinensis woodchips (AWC) were obtained from San Ho Timber Pte Ltd (Singapore) and used as received, with particle sizes ranging from 2 to 10 mm. Sugarcane bagasse (SC) was collected from local juice vendors across Singapore, rinsed with deionized water to remove surface contamination, oven dried at 60 °C for 48 h, and then mechanically milled to produce fibers of approximately 3–5 mm in length. This particle size range was selected to provide a balance between nutrient availability and structural reinforcement during mycelial growth.

Cassava pulp was obtained from a starch-processing plant in Bandung, Indonesia. Upon collection, it was stored at -20 °C immediately to prevent fermentation and microbial degradation, and thawed to ambient temperature (25 ± 2 °C) before use. Cassava pulp is a by-product of starch extraction, and is typically discarded or burned in open fields near processing facilities. Its high moisture content and residual starch make it a readily accessible nutrient source for fungal growth and colonization.

The initial moisture contents of the raw substrates, prior to sterilization, were 8–10% for AWC, 6–8% for SC, and 70–75% for cassava pulp, as measured gravimetrically by oven drying at 105 °C for 24 h.

### Fungal strain and spawn preparation

The fungal strain used in this study was *G. lucidum*, a white-rot basidiomycete capable of simultaneously decomposing lignin, cellulose, and hemicellulose. This species was selected for its ability to generate dense, cohesive mycelial networks that enhance inter-particle bonding in lignocellulosic substrates^64^. The strain was obtained from the Research Group at Republic Polytechnic (Singapore) and maintained on potato dextrose agar (PDA) plates at 4 °C until use. Prior to spawn preparation, the culture was subcultured onto fresh PDA plates, which were made every 3 months, under sterile conditions and incubated at 25 ± 2 °C for 7 days to obtain actively growing mycelium. PDA was prepared by dissolving 20 g agar, 20 g dextrose, 6 g potato extract and 0.1 g chloramphenicol (Sigma Aldrich) in 1 L of distilled water and bringing the solution to a boil. The medium was then sterilized by autoclaving at 121 °C for 15 min, cooled to 40–45 °C, and poured into sterile Petri dishes.

A liquid mycelial inoculum was prepared by aseptically transferring approximately 10 mm agar plugs from the actively growing margin of 7-day-old PDA cultures into sterile potato dextrose broth (PDB), prepared by dissolving 20 g of dextrose and 4 g of potato extract in 1 L of distilled water, bringing the solution to a boil, sterilizing it by autoclaving at 121 °C for 15 min, and allowing it to cool to room temperature before inoculation. The cultures were incubated in an orbital shaking incubator (New Brunswick™ Innova® 40, Eppendorf, Germany) at 25 ± 2 °C and 50 rpm for 7 days to produce a homogeneous liquid mycelial culture. Immediately before spawn inoculation, the liquid culture was homogenized using a sterile laboratory homogenizer to obtain a uniform mycelial suspension for inoculation.

Rice grains were used as the carrier material for spawn production, following established procedures for *G. lucidum* cultivation^48^. Rice was soaked in distilled water for 12 h, then drained and air-dried to achieve a moisture content of approximately 45%. To maintain a slightly alkaline pH that suppresses bacterial contamination and promotes fungal enzyme activity, 2 wt.% calcium carbonate (CaCO_3_) was mixed homogenously into the hydrated grains.

Approximately 200 g of the prepared rice was loaded into 500 ml autoclavable glass jars, sealed with cotton-plugged lids, and sterilised by autoclaving at 121 °C for 30 min (Tommy SX-700, Digital Biology, Japan). After cooling to room temperature under aseptic conditions, each jar was inoculated with 5 ml of homogenized liquid mycelial inoculum. Because the inoculum consisted of homogenized hyphal fragments rather than discrete fungal cells, colony-forming units (CFU mL⁻^1^) were not determined. Instead, inoculum consistency was ensured by maintaining identical culture age, growth conditions, inoculum volume, and homogenization procedures for all batches. The jars were incubated at 25 ± 2 °C and 70 ± 5% relative humidity for 14 days, with gentle shaking performed every 3 days to promote uniform colonization.

Complete colonization was indicated by the formation of a continuous white mycelial network covering all grains. The fully colonized spawn was stored at 4 °C and used within 30 days. All procedures were performed under sterile conditions in a Class II laminar-flow cabinet that was sterilized by ultraviolet irradiation for 30 min before and after use to minimize the risk of contamination.

### Substrate inoculation and incubation

The initial moisture contents of cassava pulp, SC, and AWC were determined prior to formulation. The substrate components were then weighed according to the formulations presented in Table 2 on a dry weight basis and thoroughly homogenized. Water was subsequently added to each formulation to achieve a moisture content of 60%. The formulations were designed to maintain a comparable total dry mass of the solid components while evaluating the effect of cassava supplementation.

**Table 2:** Formulations for substrate mixtures used for mycelium composite production with and without Cassava. All proportions are expressed on a dry weight basis (wt.%).

Material	AWC	SC	AWC + Cassava	SC + Cassava
	Composition (wt.%)			
AWC	74	0	38	0
SC	0	74	0	38
Cassava	0	0	45	45
Wheat Bran	24	24	12	12
CaCO_3_	2	2	5	5

In cassava-containing formulations, a portion of the base substrate (SC or AWC) was replaced with cassava pulp to maintain a consistent overall solids content. The proportion of wheat bran, which served as a nutrient supplement, was correspondingly reduced to avoid excessive nutrient enrichment following cassava addition. The CaCO₃ content was slightly increased in cassava-containing formulations to account for the acidity of the cassava pulp and to maintain favourable pH conditions for mycelial growth and colonization.

Approximately 200 g of each wet substrate formulation was then transferred into autoclavable polypropylene cultivation bags. Each bag was sealed using a cotton plug wrapped in sterile gauze, allowing limited air exchange during incubation. Sterilization was carried out at 121 °C for 30 min using an autoclave (Tomy SX-700, Digital Biology, Japan). This step minimized microbial contamination and was applied consistently across all formulations to enable reproducible fungal growth.

After sterilization, the bags were cooled to room temperature (25 ± 2 °C) in a Class II laminar-flow cabinet disinfected with 70% ethanol and exposed to ultraviolet light for 30 min before use. Each bag was then aseptically inoculated with 40 g of *G. lucidum* rice-grain spawn, corresponding to approximately 5% of the substrate dry mass. The spawn was distributed uniformly throughout the substrate by gentle manual mixing to promote homogeneous mycelial colonization throughout the bulk material.

The inoculated bags were resealed with sterile cotton plugs and placed in a controlled-environment growth chamber maintained at 25 ± 2 °C and a relative humidity of at least 65%. Continuous air circulation was provided to prevent local carbon dioxide accumulation and to maintain uniform environmental conditions. Incubation was carried out for 5–8 days, during which the fungal mycelium spread rapidly through the substrate matrix. Colonization was considered complete when a continuous, white mycelial layer fully covered the surface as assessed by visual observation. Bags showing signs of contamination were discarded immediately to prevent cross-infection.

**Fig. 5:**
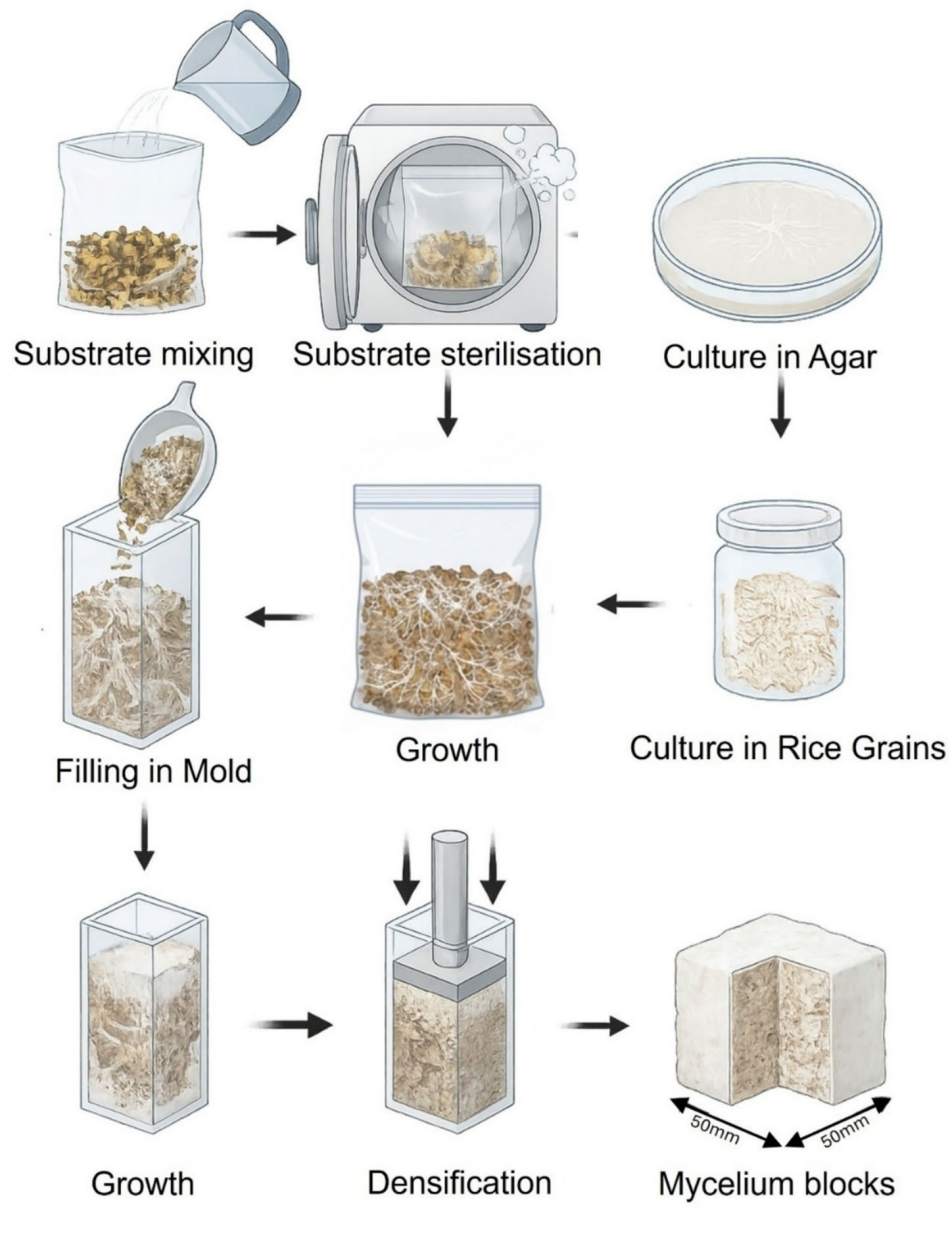
Schematic illustration of the fabrication process for mycelium-based composites. The substrate components are first mixed with water to achieve a moisture content of 60%, followed by sterilization in an autoclave at 121 °C for 30 min. In parallel, *Ganoderma lucidum* is cultured on PDA and subsequently propagated on rice grains to prepare the spawn. After cooling, the sterilized substrate is inoculated with the rice grain spawn and incubated in cultivation bags at 25 ± 2 °C and a relative humidity of at least 65% for 5–8 days until surface colonization is achieved. The colonized substrate is subsequently transferred into molds of 50 × 50 × 100 mm dimensions and incubated for a further 3–5 days to promote recolonization and consolidation. The composites are subsequently mechanically densified and maintained under controlled incubation for a further 4–10 days before demolding. The resulting material forms a rigid mycelium composite block with a continuous dense outer mycelial skin.

Once a continuous white mycelial layer covered the substrate surface, it was transferred to the molding stage for composite formation. Colonization was assessed by visual inspection based on the formation of a continuous mycelial layer across the exposed substrate surface. At this stage, *G. lucidum* formed a dense, cotton-white hyphal network with fine, branching filaments, indicating advanced colonization and effective binding of the substrate particles. The subsequent molding, mid-growth compression, and post-compression incubation steps are illustrated schematically in Fig. 5 and described in the following section.

### Fabrication of mycelium-based composites

After the initial incubation, the colonized substrates were gently disaggregated under sterile conditions to break up compacted clumps and obtain a uniform bulk consistency. This step promotes homogeneous regrowth of the fungal mycelial network during subsequent shaping and reduces the risk of uncolonized void formation inside the composite body. The loose, mycelium-based material was then manually packed into acrylic molds with dimensions of 50 × 50 × 100 mm, corresponding to the to the sample dimensions required for compression testing.

Each mold was filled progressively in layers of approximately 2–3 cm, with light manual compaction applied between layers to minimize trapped air and reduce internal voids. The filled molds were then covered with perforated lids lined with sterile filter paper to maintain sterility while allowing limited air exchange. The samples were placed in a controlled-environment growth chamber maintained at 25 ± 2 °C and a relative humidity of at least 65% for an additional 3–5 days. During this phase, the mycelium rapidly recolonized the disturbed matrix and re-established hyphal continuity across particle interfaces. Visual monitoring confirmed the formation of a continuous, cotton-white mycelial layer on the exposed surfaces.

To improve packing density and enhance the mechanical integrity of the composites, the partially colonized blocks were subsequently compressed to approximately 50%of their initial height using custom-fabricated stainless-steel clamps. Compression was maintained for 3–5 days under the same incubation conditions, allowing continued fungal growth before the clamps were removed.

After releasing the clamps, the partially consolidated blocks were incubated for a further 4–10 days, during which the mycelium formed a dense, continuous outer skin. This surface layer functions as a natural bio-coating, protecting the underlying matrix from contamination and moisture loss. Air circulation within the chamber was maintained continuously to prevent CO₂ accumulation and surface condensation. Mycelial maturity was determined visually by the formation of a uniform, white surface, indicating complete colonization and matrix consolidation.

Upon reaching full growth, the mycelium-based composites exhibited a firm, sponge-like texture with cohesive particle bonding and visible hyphal bridges throughout the cross-section. These specimens were then dried and subjected to physical and mechanical characterization as described in the following section.

### Post-growth drying and material characterization

After completing mycelial colonization, the mycelium-based composites were removed from the molds and prepared for drying. This post-growth treatment was applied to terminate fungal metabolic activity, remove residual moisture, and stabilize the internal microstructure.

Drying was conducted in a laboratory convection oven (Qingdao Guosen-1800, China) at 70 °C for 48–72 h, or until a constant mass was achieved. The selected drying temperature was sufficient to inactivate the fungal biomass while avoiding thermal degradation of the lignocellulosic substrate and chitinous mycelial matrix, as demonstrated in prior studies^45^. Samples were placed on stainless-steel mesh trays to ensure uniform air circulation, and sample mass was recorded at 12 h intervals using a precision analytical balance (Shimadzu UW series, Japan).

Before and after drying, the dimensions of each specimen were measured using a digital Vernier calliper (Mitutoyo, Japan, accuracy ± 0.01 mm) to quantify shrinkage and volumetric change. The geometric volume was calculated from the average length, width, and height of each sample, and the bulk density (ρ) was determined using Eq. 1:

1 $$\rho =\frac{{m}_{d}}{{V}_{d}}$$

where *m*_*d*_​ is the dry mass (kg) and *V*_*d*_​ is the corresponding dry volume (m^3^). Three independent samples (n = 3) were tested for each formulation.

Visual assessments were performed after drying to evaluate surface uniformity, color, and overall integrity. The fully dried composites exhibited rigid, lightweight structures with visible hyphal bonding and minimal warping or surface defects, consistent with the characteristics of well-grown *Ganoderma*-based mycelium composite materials. These stabilized samples were subsequently used for mechanical testing and microstructural characterization.

### Porosity measurement

Porosity was quantified to assess the internal void structure and degree of compactness of the mycelium-based composites. Porosity is a key parameter influencing mechanical strength, moisture transport, and dimensional stability. The total porosity was determined from the relationship between the bulk density and the skeletal density, with the latter measured using a helium gas pycnometer based on the gas displacement principle.

Helium gas pycnometry determines the true skeletal volume of the solid phase by measuring the pressure change that occurs when an inert gas (in this case, helium) is expanded from a calibrated reference chamber into a chamber containing the sample. Because helium atoms are small and chemically inert, they penetrate fine surface-accessible pores but do not enter the closed internal voids, allowing accurate determination of the solid-phase (skeletal) volume. This method is particularly suitable for highly porous bio-based composites, as it avoids liquid absorption, swelling effects and structural alteration that can occur with immersion-based porosity measurement methods.

Before measurement, each specimen was oven-dried at 60 °C for 12 h to remove residual moisture and then weighed to determine its dry mass. The sample was placed in the helium pycnometer chamber, and after thermal equilibration, a controlled helium pressure pulse was released from the reference chamber. The equilibrium pressures in both chambers were recorded automatically, and the sample skeletal volume (*V*_*s*_​) was calculated by the instrument software using the ideal gas law. The skeletal density (*ρ*_*skeletal*_​) was determined using Eq. 2.

2 $${\rho}_{skeletal}=\frac{{m}_{d}}{{V}_{s}}$$

where *m*_*d*_​ is the dry mass of the specimen.

The bulk density (*ρ*_*bulk*_​) was obtained independently from the dry mass and geometric volume measured using a digital vernier calliper (accuracy ± 0.01 mm). The total porosity (P) was calculated using Eq. 3:

3 $$\mathrm{P}= \left(1-\frac{{\rho}_{bulk}}{{\rho}_{skeletal}}\right)\times 100$$

Three independent samples (n = 3) were tested for each substrate formulation. Between consecutive measurements, specimens were re-dried at 60 °C to remove any absorbed moisture and ensure consistent measurement conditions. This gas-displacement method provides a highly reliable and reproducible measure of total porosity and internal compactness, parameters that are directly linked to the mechanical performance and microstructural connectivity discussed in subsequent sections.

### Mechanical testing

The compressive strength, elastic modulus in compression and corresponding stress–strain behaviour of the mycelium-based composites were determined to evaluate their stiffness, load-bearing capacity, and deformation mechanisms. Compression testing was selected as the primary mechanical characterisation method because mycelium-based composites exhibit a cellular, foam-like structure and are commonly evaluated under compressive loading for applications such as insulation panels and light-weight structural cores.

Tests were performed using a universal testing machine (Shimadzu AG-IC, Japan) equipped with a 100 kN load cell and flat, polished compression platens. The testing procedure followed ASTM D3574-17, which specifies the determination of compressive properties for cellular and foam-like materials. Prior to testing, all specimens were conditioned at 23 ± 2 °C and 50 ± 5% relative humidity for a minimum of 12 h, to ensure consistent moisture equilibrium.

Each specimen was centrally aligned between the compression platens and tested under displacement-controlled loading at a constant crosshead speed of 10 mm/min. Load and displacement were continuously recorded at a sampling rate of 10 Hz. The nominal compressive stress (σ) and strain (ε) were calculated using Eqs. 4 and 5 respectively:

4 $$\sigma =\frac{F}{{A}_{0}}$$

5 $$\varepsilon =\frac{\Delta h}{{h}_{0}}$$

where *F* is the applied load (N), *A*_*0*_​ is the initial cross-sectional area (*m*^*2*^), *h*_*0*​_ is the initial specimen height (m), and *Δh* is the measured displacement (m).

The elastic modulus in compression (*E*_*c*_​) was determined from the slope of the initial linear portion of the stress–strain curve, typically between 0.5 and 2% strain, where the material response is approximately linear and reversible. A least-squares linear regression was applied to the selected data points to calculate the modulus using Eq. 6, where ${\Delta}_{\sigma }$ represents the change in stress and ${\Delta}_{\varepsilon }$ the corresponding change in strain within the selected linear elastic region:

6 $${E}_{c}=\frac{{\Delta}_{\sigma }}{{\Delta}_{\varepsilon }}$$

The compressive strength at 5% strain was defined as the stress corresponding to 5% deformation, representing the onset of localized cell collapse before densification, consistent with the strain limits recommended in ASTM D3574-17 for flexible cellular materials.

Three independent specimens (n = 3) were prepared and tested for each substrate formulation, and the mean values with corresponding standard deviations were reported. During testing, deformation behaviour was visually monitored to identify the transition from the initial elastic regime to the plateau region. The stress–strain response typically displayed an initial linear elastic regime, followed by a plateau associated with hyphal-fiber network buckling and progressive densification at higher strains.

The measured elastic modulus and compressive stress values were subsequently correlated with porosity to interpret how substrate type and internal structure influence the mechanical performance of the mycelium-based composites.

### Microstructural characterization by scanning electron microscopy (SEM)

The microstructure of the mycelium-based composites was examined using SEM to visualize hyphal distribution, substrate-hypha interactions, and pore morphology within the dried composites. Observations were performed using a JEOL JSM-5500LV electron microscope (Japan) operated under high-vacuum mode at an accelerating voltage of 3.0 kV and a working distance of 15 mm.

Before imaging, small sections (approximately 10 × 10 × 5 mm) were cut from representative regions of each composite, including both surface (skin) and core. The samples were mounted on aluminum stubs using carbon adhesive tape and sputter-coated with a thin carbon layer for 20 s using a Pelco SC-6 carbon coater (Ted Pella Inc., USA) to minimize surface charging during imaging.

A low accelerating voltage was chosen to minimize electron-beam penetration and charging effects in the non-conductive organic matrix while maintaining sufficient surface resolution. Images were collected at 1500 × magnification to ensure consistent comparison of the hyphal networks and substrate morphology between formulations. Surface images were used to assess the formation of continuous mycelial films and bridging filaments, and core images were used for hyphal diameter measurements and evaluation of the internal substrate-hypha morphology.

Qualitative comparisons were made across samples prepared from different substrate types to assess the influence of substrate morphology and nutrient composition on hyphal density and bonding behaviour. These micrographs complemented the mechanical and porosity measurements, providing visual evidence of how the fungal matrix contributes to load transfer and structural reinforcement within the composite architecture.

Hyphal diameter distributions were obtained by manually measuring 250 individual hyphae diameters from each SEM image using the ImageJ software. Fungal hyphae were distinguished from the SC and AWC substrate fibers in SEM images based on their characteristic morphology and size. Hyphae appeared as slender, smooth, cylindrical filaments with relatively uniform diameters in the micrometer range. In contrast, the SC and AWC substrate fibers were substantially larger, exhibited irregular geometries, and possessed rough surface textures. These characteristics enabled reliable identification of hyphae for diameter measurements.

## Data Availability

The data that support the findings of this study are available from the corresponding author upon request.
